# Monolayer graphene/platinum-modified 3D origami microfluidic paper-based biosensor for smartphone-assisted biomarkers detection

**DOI:** 10.5599/admet.2833

**Published:** 2025-07-20

**Authors:** Arda Fridua Putra, Annisa Septyana Ningrum, Suyanto, Vania Mitha Pratiwi, Muhammad Yusuf Hakim Widianto, Irkham, Wulan Tri Wahyuni, Isnaini Rahmawati, Fu-Ming Wang, Chi-Hsien Huang, Ruri Agung Wahyuono

**Affiliations:** 1 Department of Engineering Physics, Institut Teknologi Sepuluh Nopember, Surabaya 60111, Indonesia; 2 Department of Materials Engineering, Institut Teknologi Sepuluh Nopember, Surabaya 60111, Indonesia; 3 Department of Mathematics, Institut Teknologi Sepuluh Nopember, Surabaya 60111, Indonesia; 4 Department of Chemistry, University of Padjadjaran, Sumedang 45363, Indonesia; 5 Department of Chemistry, Institut Pertanian Bogor (IPB) University, Bogor 16680, Indonesia; 6 Department of Chemistry, University of Indonesia, Depok 16424, Indonesia; 7 Graduate Institute of Applied Science and Technology, National Taiwan University of Science and Technology, Taipei 10607, Taiwan; 8 Graduate Institute of Energy and Sustainability Technology, National Taiwan University of Science and Technology, Taipei 10607, Taiwan; 9 Department of Materials Engineering, Ming Chi University of Technology, New Taipei City 24031, Taiwan

**Keywords:** Colorimetry, diagnostic kit, dopamine, NADH, nanocatalyst

## Abstract

**Background and purpose:**

Imbalances in biomarkers such as dopamine and NADH are linked to neurological and metabolic disorders, including Parkinson’s disease, depression, and stroke, underscoring the need for rapid and accessible diagnostics. This study presents a smartphone-assisted, 3D origami microfluidic paper-based analytical device (μPAD) modified with photochemically synthesized graphene/platinum (G/Pt) nanocatalysts for multiplex colorimetric detection of dopamine and NADH.

**Experimental approach:**

G/Pt catalysts were prepared using 2.5 to 10 mM Pt precursors under UV irradiation. μPADs were laser-printed on commercial-grade filter paper, patterned, and folded into three layers of 3D Origami.

**Key results:**

The optimized 10 mM G/Pt catalyst significantly improved reaction rates (18× faster), leading to a rapid detection time constant of 6.69 and 4.59 s for dopamine and NADH, respectively. Furthermore, the utilization of 10 mM G/Pt catalyst increased colour intensity (2.48×) on the μPAD platform. An application for smartphones integrated with an image processing algorithm was developed using Kotlin to enable automatic quantification of colorimetric signals from saturation and hue channels for dopamine and NADH, respectively. The detection exhibited the lowest mean absolute percentage errors of 0.52 and 0.07 % as well as a limit of detection of 0.56 and 0.99 mM for dopamine and NADH, respectively.

**Conclusion:**

The 3D origami structure facilitates efficient fluid handling and multiplex detection, while the nanocatalyst modification improves pore infiltration and sensitivity. This work demonstrates, for the first time, a cost-effective, portable, and high-performance biosensor for dual biomarker detection, offering substantial promise for point-of-care diagnostics in neurological and metabolic health monitoring.

## Introduction

Health plays a vital role in overall human well-being, and its assessment often relies on the analysis of specific biological compounds known as biomarkers. Disruptions in these biomarkers, such as dopamine deficiencies, have been associated with neurological and psychiatric disorders, including schizophrenia, depression, and Parkinson’s disease [1]. Similarly, elevated levels of NADH have been linked to various health issues, including depression and stroke [2]. These associations highlight the growing demand for diagnostic tools that are not only affordable and user-friendly but also portable. In response, biosensors have been developed for efficient biomarker detection. Among the emerging technologies, microfluidic paper-based analytical devices (μPADs) have gained significant attention due to their low production cost, biodegradability, and ease of fabrication [3]. The functionality of μPADs relies on patterned hydrophobic barriers that guide fluid flow, typically created through techniques such as photolithography, wax printing, and inkjet printing [4]. To improve their sensitivity and efficiency, 3D μPADs have been introduced, offering advantages such as minimized sample loss and faster analytical response [5]. One of the most promising approaches to fabricating these 3D devices is the origami method, which simplifies the process to just printing and folding, making it highly practical for widespread application [6].

Biomarker detection and quantification in 3D μPADs can be achieved through several analytical techniques, each offering distinct advantages. One widely used method is chemiluminescence, which detects light emitted from enzyme-catalysed reactions, such as glucose oxidase, to measure glucose levels in biological samples [6]. Another technique involves electrochemical methods, utilizing amperometry detection to monitor glucose through the current generated during the oxidation process [7]. Electrochemical sensors and biosensors have demonstrated broad applicability in detecting biomarkers such as dopamine, ascorbic acid, and uric acid, particularly in complex matrices like human urine and plasma. These sensors offer high sensitivity, rapid response, and selectivity using various modifiers, including electrochemically reduced graphene oxide (ERGO), polyCoTAPc, multi-walled carbon nanotubes (MWCNTs), molecularly imprinted polymers (MIP), and 2D MXene nanoplatelets [8]. Additionally, colourimetry is a reliable and widely used method for detecting biomarkers. This approach involves a reaction between the analyte and a reagent, resulting in a visible colour change upon interaction [9]. Due to its simplicity, rapid detection, accuracy, sensitivity, and stability, colourimetry is a preferred technique for biosensors [10] Moreover, the results from colorimetric analyses can be interpreted visually or enhanced through image analysis and digital tools to enable precise quantitative assessment. Smartphones can serve as optical devices for biosensing, offering improved accuracy and linearity by either capturing images directly or processing them to display results. Currently, image analysis and biomarker quantification are often performed manually using open-source software such as ImageJ [11]. However, dedicated smartphone applications that fully automate the analysis process remain limited. Colorimetric changes can be interpreted through various colour models, including RGB (Red, Green, Blue), HSV (Hue, Saturation, Value), and grayscale. Among these, RGB and grayscale are the most commonly used channels for biomarker detection, although recent studies have also demonstrated the effectiveness of the HSV model in enhancing the accuracy of colorimetric analysis [12]. The integration of these features into smartphone-based platforms holds great potential for advancing point-of-care diagnostics by combining simplicity, precision, and accessibility.

It is also crucial to note that the sensitivity and linearity of the biosensor can be enhanced by modifying the μPAD using nanomaterials [13]. Metal nanomaterials such as platinum, palladium, gold, and silver are widely utilized as catalysts to increase the sensitivity of colorimetric biosensors [14]. Previous studies determined that the catalytic activity for H_2_O_2_ decomposition follows the order Au < Ag < Pt, based on adsorption and activation energy calculations [15,16]. However, Pt nanoparticles tend to aggregate in solution, leading to a decrease in catalytic activity. Anchoring metal particles onto a specialized carrier is considered an effective method to prevent aggregation and maintain the catalytic activity of the nanoparticles [17]. In this context, compositing metal nanoparticles with graphene has been proven to enhance the change in colour intensity, where graphene-metal nanocomposites exhibit three times higher catalytic activity in detecting neurotransmitters [18]. Geometrically, graphene-Pt (G/Pt) nanocomposites possess a surface area six times larger than that of Pt nanoparticles, thereby increasing the loading capacity of analytes and reagents on μPAD paper [16].

In this work, a biomarker detection kit for dopamine and NADH was developed. Laser-printed 3D origami μPAD were modified with graphene-Platinum (G/Pt) nanocomposite as a catalyst. The Graphene-Platinum nanocomposites were synthesized through a photochemical method, an innovative approach that is quick, straightforward, and produces more uniform nanoparticles. Furthermore, a portable detection chamber was fabricated to aid in the detection of dopamine and NADH using the modified μPAD. Additionally, an Android smartphone application was developed using the Kotlin language to facilitate the automatic measurement of biomarkers. The developed G/Pt and μPADs were investigated using scanning electron microscopy (SEM), transmission electron microscopy (TEM), energy-dispersive X-ray spectroscopy (EDX), X-ray photoelectron spectroscopy (XPS), Fourier-transform infrared spectroscopy (FTIR), contact angle measurement, and atomic force microscopy (AFM).

## Experimental

### Materials and components

The catalyst precursors were monolayer graphene (Ossila, UK), hexachloroplatinic acid (H_2_PtCl_6_, Smart Lab Indonesia), potassium iodide (KI, Merck), and polyvinylpyrrolidone K30 (PVP, BASF). The biomarkers were dopamine hydrochloride and grade II nicotinamide adenine dinucleotide hydrogen (NADH) (both from Merck). The reagents for colorimetric detection were (FeCl_3_), phenanthroline, 2,4-dinitrophenylhydrazine (DNP), potassium periodate (PPI), sodium hydroxide (NaOH), and resazurin salt (All from Merck). Colorimetric detection chamber utilized 10-lumen 2835 SMD LED, purchased from a local supplier, as light sources. Commercial grade lab filter papers with a pore size of 15 to 20 μm and a thickness of 200 μm were purchased from a local supplier.

### Synthesis of G/Pt nanocomposite

Synthesis of monolayer graphene/platinum (G/Pt) nanocomposite was conducted by photochemical reduction using H_2_PtCl_6_ as precursor and KI as reducing agent under constant ultraviolet (UV) irradiation [16,19]. In this work, the concentration of H_2_PtCl_6_ varied from 2.5 to 10 mM and was mixed with KI in a mole’s ratio of 1:4. Subsequently, 3.75 grams of PVP were added in 10 mL of distilled water, followed by the addition of 5 mL of monolayer graphene with a concentration of 2 mg ml^-1^. The mixture was thoroughly homogenized before being exposed to 15 W UV light (wavelength: 254 nm, intensity: 30 μW cm^-2^) for 2 hours, with irradiation applied from the top and without agitation. The resulting suspension was centrifuged at 4000 rpm for 45 minutes, washed with ethanol, dried, and finally redispersed in 5 mL of distilled water. The G/Pt nanocomposites were labelled as G/Pt (2.5), G/Pt (5.0), G/Pt (7.5), and G/Pt (10.0), corresponding to the initial concentrations (in mM) of the platinum precursor used during synthesis.

### Characterization of G/Pt nanocomposite

The as-synthesized G/Pt nanocomposites were characterized using low-resolution transmission electron microscopy (TEM), scanning electron microscopy and energy dispersive X-ray (SEM-EDX), UV-vis absorption and Fourier transform infrared (FTIR) spectroscopy to determine the morphology and characteristics of the electronic nature and functional groups. The SEM-EDX analysis was performed using an FEI Inspect S50 at an accelerating voltage of 40 kV on an indium-doped SnO_2_-SiO_2_ (ITO) substrate, while TEM images were collected using an HT7700 (Hitachi) at an accelerating voltage of 120 kV. UV-vis absorption (200 to 800 nm) and IR transmission (4000 to 400 cm^-1^) spectra were collected using ThermoScientific Genesys 150 and ThermoNicolet iS50 spectrometers, respectively. The compositions of the samples were measured using X-ray photoelectron spectroscopy (XPS, VG ESCA Scientific Theta Probe) with a monochromated Al-Ka source. In addition, G/Pt was dropped onto the detection zone of μPAD paper to modify its surface properties. The μPAD surface was then analysed using atomic force microscopy (AFM, Brucker Nanoscan) to examine the topography and the surface roughness. The contact angle test was also conducted to determine its hydrophobicity. Additional UV-vis spectroscopy characterization was used to verify colour changes produced during analyte detection.

### Computational study of G/Pt nanocomposite

A computational study was conducted to investigate the potential cluster formation of Pt nanoparticles on a graphene matrix. Initial studies of the computational model focused on a single Pt atom positioned at various sites on the graphene layer: the midpoint of a C-C bond, directly above a single carbon atom, and the centre of a hexagonal graphene site [20]. The PHASE/0 code was employed, utilizing spin-polarized density functional theory to model the G/Pt layers. This code, designed for low-dimensional materials and large-scale calculations, was instrumental in optimizing the lattice constant of graphene. A slab model was used to simulate G/Pt layers, incorporating a 150 nm vacuum space to prevent interlayer interactions. The Monkhorst-Pack k-point grid was set to 16×16×1, and the projector augmented wave method was applied. Subsequently, geometry optimization was performed, ensuring atomic forces were less than 8.01×10^-8^ J m^-1^ [21].

### Design and fabrication of 3D origami μPADs

**Figure 1.**
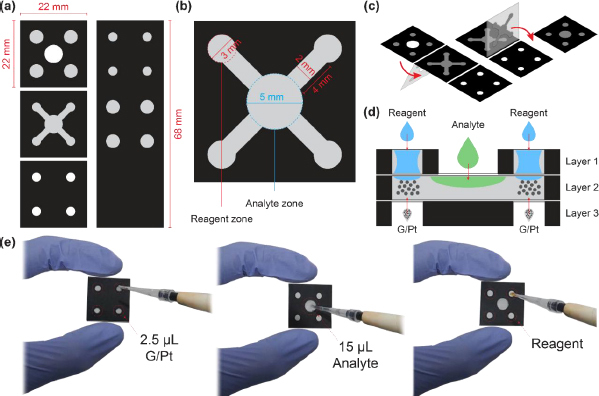
Design of 3D origami μPAD with the (a) front view, (b) size detail, (c) folding direction and (d) cross-section view, as well as (e) the preview of fabricated 3D μPAD with corresponding deposition steps

Filter papers were used for μPAD fabrication following the design in Figure 1. The μPAD main design consists of four detection zones with a diameter of 3 mm, fluid flow channels with a width of 2 mm and a length of 4 mm, and sample zones with a diameter of 5 mm.

The first layer is designed for reagents and acts as analyte inlet channels into the second layer, while the third layer is designed as an inlet for depositing the G/Pt catalyst. The analytes will then flow into the detection zone in the second layer and mix with the reagent, starting the catalytic-induced reaction and producing colour change. μPADs were fabricated with commercial-grade filter paper using a laser jet printer (HP P1102) and then heated in an oven at 155 °C for 12 minutes for an optimum hydrophobic barrier. The paper was then punched and folded into three layers to form 3D Origami.

### Design and fabrication of colorimetric detection chamber

**Figure 2.**
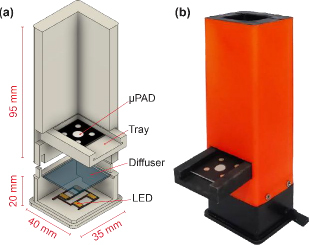
(a) The design and (b) the fabricated detection chamber

The colorimetric detection chamber was fabricated using a 3D printer following the design in Figure 2 for more accurate and controlled measurement. The detection chamber consisted of two parts, with the lower part acting as a light source and featuring four LEDs placed parallel to the μPAD detection zone. A diffuser was placed above the LED to evenly distribute the light. The LEDs were then connected to a 12 V DC adaptor as the power supply. The upper part serves as the holder for the smartphone camera, with the chamber height designed according to the camera's focal length. This design also prevents interference from ambient light, as the uniformity of light intensity affects the accuracy of reading. To ensure consistency between captured and original images, various camera settings were optimized, specifically ISO values (200, 400, and 640) and shutter speeds (1/60 and 1/45).

### Colourimetry detection

Dopamine and NADH, within the concentration range of 0.01 to 10 mM, were used as the analytes to evaluate biomarker detection, with each biomarker dissolved in PBS at pH 7.4. Stock solutions were prepared by dissolving 0.18 M FeCl_3_, 14.5 mM phenanthroline, 6.5 mM PPI, 0.8 mM DNP, and 10 M NaOH in deionized water. Resazurin was prepared by dissolving 0.8 mM of Resazurin salt in a 0.1 M Tris-HCl buffer at pH 7.4. The μPAD was first modified with G/Pt by dropping 2.5 μL of the solution into each of the detection zones. Subsequently, 15 μL of the biomarker solution was added to the μPAD. Detection using FeCl_3_ and phenanthroline was performed by adding 2.5 μL of FeCl_3_ and 2.5 μL of phenanthroline. The resazurin detection followed the same procedure, with 2.5 μL of resazurin added. For detection using DNP, 2.5 μL of PPI, 2.5 μL of DNP and 5 μL of NaOH were sequentially added. For the dynamic response test, the colour change reaction was recorded and the colour intensity of saturation and hue over time was measured using ImageJ software (https://imagej.net/). The dynamic responses of the colour channel changes were then fitted exponentially using Origin software (https://www.originlab.com/), where the best channel response was chosen to determine the optimal time constant for colour measurement.

In the static response test, the colourimetry detection was carried out using 16 variations of biomarker concentration. At the optimal time after the dropping, the colour was captured with a smartphone and then measured using ImageJ (details in Supplementary material). A calibration curve was obtained through linear regression of the colour intensity using Origin. The performance of μPAD was then evaluated by analysing the calibration curve, including linearity (*R*^2^), sensitivity (gradient), limit of detection (LOD), limit of quantification (LOQ) and sensor mean absolute percentage error (MAPE) value. LOD and LOQ were calculated by Equations (1) and (2):



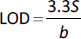

(1)




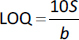

(2)


where *S* is the standard deviation of the y-residuals and y-intercepts of the linear regression line, and *b* is the slope of the calibration curve.

### Smartphone application

An application for Android smartphones was developed using Kotlin to facilitate the measurement of biomarkers. The application featured an easy-to-use menu that allowed users to capture or import images to be processed using a developed image processing algorithm with the OpenCV Android module. The biomarker reading results obtained from the calibration curves were then displayed in the application. The image processing is performed by dividing the image based on the detection zones of the μPAD, followed by thresholding and masking to obtain images of the detection zones. Subsequently, the images of the detection zones' colour intensity were measured for red (R), green (G), blue (B), and grayscale within the range of 0 to 255, while colour intensity measured in hue (H), saturation (S) and value (V) were measured within the range of 0 to 360, 0 to 100, 0 to 100, respectively. Details of the image processing algorithm and data processing were translated into a source code summarized in the Supplementary material.

## Results and discussion

### Physicochemical characteristics of G/Pt nanocomposites

**Figure 3.**
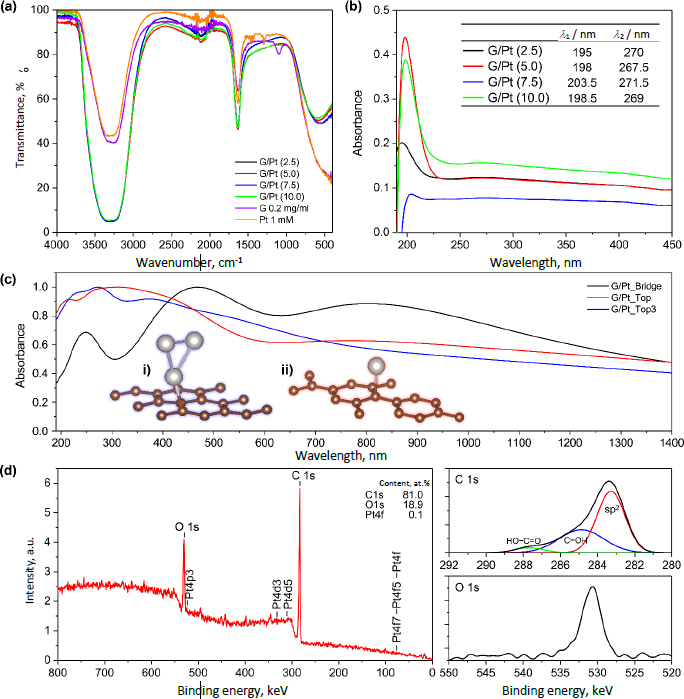
(a) IR transmittance. (b) UV-vis spectra of various G/Pt. (c) Modelled absorption spectra with the model of (i) G/Pt_Top3 and (ii) G/Pt_Top, and (d) XPS result of G/Pt (10.0)

Molecular functional groups and electronic properties of G/Pt nanocomposite as a catalyst are assessed from the IR transmittance and UV-vis absorption spectra. IR spectra, as shown in Figure 3(a), indicate a wide absorption peak between 3735 to 2765 cm^-1^ due to the presence of hydrated bonds with O-H stretching from the distilled water as the solvent. A sharp peak at 1636 cm^-1^ signifies the C=C stretching of carbon bonds in graphene [22]. The peak at 1096 cm^-1^ is only present in graphene and not in platinum or any G/Pt variations. This peak indicates the presence of C-H bending due to graphene dissolved in water. The increase in transmittance at this wavelength suggests that the C-H bonds in graphene are released, allowing the carbon to bond with platinum [23]. Another peak is observed at the wavenumber of 556 cm^-1^, indicating the presence of C-Cl bonds. Based on the peaks at wavenumbers of 1096 and 556 cm^-1^, it could be inferred that the C-H bonds in the graphene solution are released during the synthesis process, subsequently bonding with reduced platinum and chlorine.

Absorption spectra depicted in Figure 3(b) show nearly identical absorption maxima at ~270 and ~200 nm. However, the G/Pt (7.5 mM) nanocomposite does not show an absorption band at ~270 nm, and instead, an absorption peak appears at 236.5 nm. Commonly, nanoparticles exhibit absorption bands at 216 and 264 nm [24] where the absorption at 265 nm is associated with the absorption of the carbonyl bond of graphene, *i.e.* the characteristic of the π-π* transition [25]. The disappearance of the 270 nm absorption band for the G/Pt (7.5 mM) nanocomposite may indicate the band splitting in π-π* character, and the formation of a new absorption band at 236.5 nm can be caused by the interaction between the carbon in graphene and Pt nanoparticles. The UV absorbance shows a red shift of the peak absorbance (*λ*_max1_), indicating the possibility of aggregation of Pt nanoparticles.

Following the computational model, the lattice constant of graphene is determined to be 24.8 nm, belonging to the P6/mmm (D^1^_6h_) space group, with a bond length of 14.38 nm. The calculated band gap of graphene without spin-orbit coupling is below 1 eV, aligning well with previous studies [26]. After geometry optimization, it is found that the midpoint configuration is energetically favourable, with an energy 2.79×210^-17^ J for graphene lower than the top-of-carbon configuration. The Pt-C bond length for the midpoint configuration is 20.89 nm, slightly longer than the 20.10 nm bond length observed for the top-of-carbon configuration. This results in possible G/Pt models, such as single Pt_Top, Pt_Top3, and Pt_Bridge (details are provided in the Supplementary material).

The absorption spectra of the G/Pt model in various cluster configurations, as shown in Figure 3(c), exhibit distinct patterns with three characteristic absorbance peaks. Specifically, the spectra for the G/Pt_Bridge, G/Pt_Top, and G/Pt_Top3 models display peaks at approximately 250, 460, and 800 nm; 210, 320, and 800 nm; and 225, 270, and 375 nm, respectively. The UV-vis spectra of the synthesized G/Pt nanocomposites, presented in Figure 3(b), do not align with the spectrum of the G/Pt_Bridge model, notably missing peaks at around 460 and 800 nm. This suggests that the synthesized G/Pt nanocomposite is unlikely to adopt a bridge formation between Pt nanoparticles and the graphene layer, as depicted in Figure 3(b). The G/Pt_Top3 model, however, provides a plausible framework for simulating the aggregation behaviour of Pt nanoparticles on the graphene layer. As shown in Figure 3(c), the broad absorption band between 250 to 450 nm in the G/Pt_Top spectrum resolves into two distinct peaks at 270 and 375 nm in the G/Pt_Top3 model. The UV-vis spectra of the synthesized G/Pt nanocomposites show a closer similarity to the G/Pt_Top3 model than the G/Pt_Top, sharing a similar spectrum of decreasing absorbance following the initial peak around 200 nm. This observation strongly suggests the possibility of Pt nanoparticles aggregating on the graphene layer, consistent with the red shift of the primary peak observed in Figure 3(b). Elemental analysis based on the XPS data indicates a large intensity of O1s and C1s elements, which is due to the dominant fraction of the graphene monolayer. The C1s region shows shallow hydroxyl functional groups compared to the sp^2^ peak, which is consistent with other studies for Pt-modified rGO structures [27]. Pt4f peak is also observed, albeit small, as the identified Pt nanoparticles are found to account for only 0.1 % of the atomic weight.

**Figure 4.**
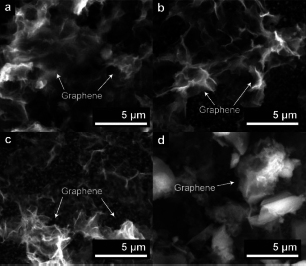
SEM results of (a) G/Pt (2.5), (b) G/Pt (5.0), (c) G/Pt (7.5), (d) G/Pt (10.0)

**Figure 5.**
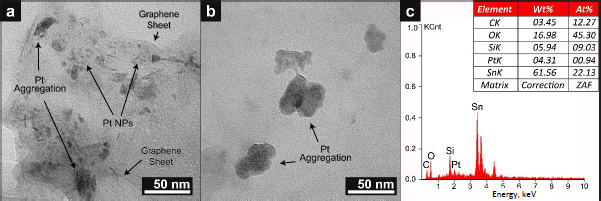
(a, b) TEM results of G/Pt (10.0) with different sampling regions. (c) EDX Pattern of G/Pt (10.0) on Indium-doped SnO_2_ glass substrate

Based on the SEM images in Figure 4, synthesized G/Pt catalyst contains clear evidence of a graphene layer. However, no Pt nanoparticles are directly observed. Further observations with TEM, shown in Figures 5(a) and 5(b), show a transparent thin sheet of graphene monolayers with uneven distribution of Pt nanoparticles in various cloud-like aggregate forms.

The largest observable particle size is estimated to be ~50 nm. This aggregation can either be caused by an inadequate amount of PVP or insufficient energy from the light source during synthesis [28]. PVP plays a function of capping agent and of stabilizer in metal colloids by covering and dispersing nanoparticles during synthesis, therefore preventing aggregation [28]. Elemental composition analysis is performed by EDX, as shown in Figure 5(c). Silicon and tin are present in the graph because ITO glass serves as the medium. The EDX result reveals the presence of Platinum, albeit in small amounts. This result is in good agreement with the XPS spectrum, indicating that Pt nanoparticles are successfully synthesized.

### Surface characteristics of G/Pt-modified μPAD

**Figure 6.**
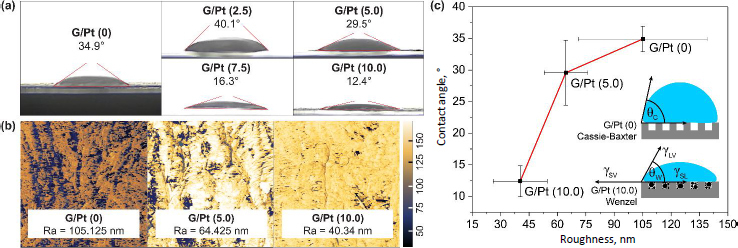
(a) Contact angle. (b) AFM test results of blank, and G/Pt-modified μPAD, (c) Correlation of the surface roughness and the contact angle of G/Pt-modified μPAD

Hydrophilicity is one of the important properties in μPADs, as it ensures unobstructed fluid permeation through the paper substrate. As shown in Figure 6(a), increasing the platinum (Pt) concentration in the G/Pt nanocomposite significantly enhances the hydrophilicity of the μPAD, demonstrated by a reduction in the contact angle from 34.9 to 12.4°. This indicates that the formed G/Pt nanocomposite was small enough to fill the pores in the paper substrate, resulting in a smoother paper surface and lower surface tension. AFM characterization in Figure 6(b) reveals a rough topography with numerous peaks on the blank paper, whereas the paper with G/Pt appears much flatter or smoother. The G-Pt modification plausibly fills the surface pore structure, leading to a reduced surface roughness.

The surface roughness (*R*_a_) for the μPAD decreases as G/Pt concentration increases from 105.125 to 40.34 nm. This indicates that higher Pt concentrations result in a smoother surface, hence turning a more hydrophobic surface into hydrophilic following the Wenzel and the Casey-Baxter model, respectively, which is formulated by Equations (3) and (4) [29]:



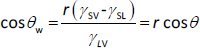

(3)


where *θ*_w_ is the Wenzel contact angle between the liquid and the solid on the rough surface, *γ*_SV_, *γ*_SL_ and *γ*_LV_ are the surface tensions between the solid-gas, solid-liquid, and liquid-gas contact surfaces, respectively, while *r* is the ratio of the actual contact area of the solid to the projected area of the contact area of the solid-liquid interface.



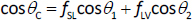

(4)


where *f*_SL_ and *f*_LV_ constitute the ratio of the solid-liquid and the liquid-gas contact surface to the apparent total contact area, respectively, while *θ*_1_ and *θ*_2_ are the liquid contact angles on the ideal smooth solid and ideal air surface, respectively. These results align with the contact angle measurements shown in Figure 6(c), where an increase in Pt concentration leads to a more hydrophilic surface, resulting from a reduction in the surface roughness ratio, which in turn decreases the angle.

### Neurotransmitter detection

**Figure 7.**
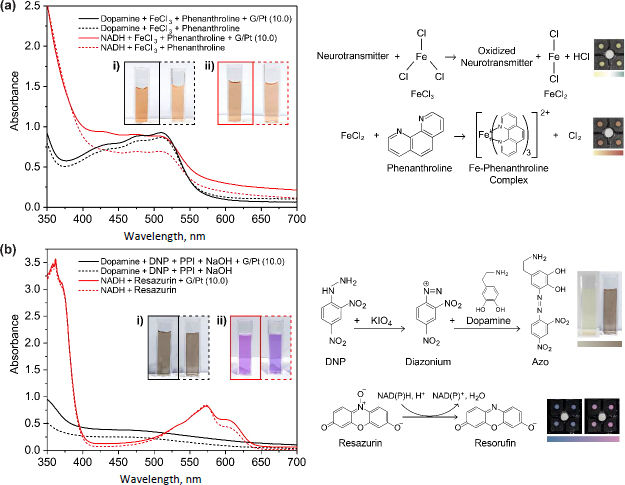
Absorbance spectrum and the colour produced by (i) dopamine and (ii) NADH using (a) FeCl_3_ + phenanthroline and (b) using DNP and resazurin with (left) and without the addition (right) of G/Pt

The absorbance spectra of dopamine and NADH during reaction with and without the addition of G/Pt can be seen in Figure 7(a). Dopamine and NADH react with FeCl_3_, forming Fe^2+^ that subsequently react with phenanthroline to form the tris(1,10-phenanthroline) iron(II) complex. During the addition of FeCl_3_, the ortho hydroxyl of dopamine reduces Fe^3+^ ions in FeCl_3_ into Fe^2+^ to form a colour change, depending on the amount of reduced Fe^3+^, from yellow to green. Subsequently, phenanthroline is added to form the Fe-phenanthroline complex, producing a colour range of pink to reddish-orange and absorbing the light at ~510 nm [30]. For NADH, there are two colorimetric reactions: the oxidation of NADH to NAD^+^, producing a brownish colour, and the reduction of FeCl_3_ to Fe^2+^, producing a yellow-green colour, therefore resulting in two colour changes. The detection of dopamine using DNP and NADH using resazurin follows the reaction mechanism illustrated in Figure 7(b). Initially, DNP is oxidized by potassium periodate (PPI), resulting in the formation of a pale yellow diazonium ion. This ion is highly reactive with compounds containing phenol groups, such as dopamine. Upon the addition of dopamine, the diazonium ion undergoes a reaction to form azo compounds, which shift in colour from reddish to brown when NaOH is introduced [31]. NADH detection utilizes the mechanism of NADH oxidation in which a hydrogen ion is released and used to reduce the blue-coloured resazurin, resulting in the formation of NAD^+^ and resorufin, exhibiting a magenta-pink colour [32].

Optimization of camera configuration prior to detection showed that the combination of 1/60 and 1/45 with ISO 200 produced the best results with digital colour intensities of 67 to 223 and 73 to 234, respectively. Standard deviation values were also relatively low compared to other configurations, 27.18 and 27.56. Comparing these configurations, the combination of 1/60 fs shutter speed and ISO 200 was optimal for capturing the images. Subsequently, a test image was taken using a smartphone camera with the following settings: focus at 0.1, ISO 200, shutter speed 1/60, and white balance (WB) set to 4400K. The resulting image showed uniform light exposure, as verified by a heatmap analysis, with no signs of overexposure from the LED illumination (see Supplementary material). Therefore, the lighting and camera configuration were optimal for the colourimetry test.

**Figure 8.**
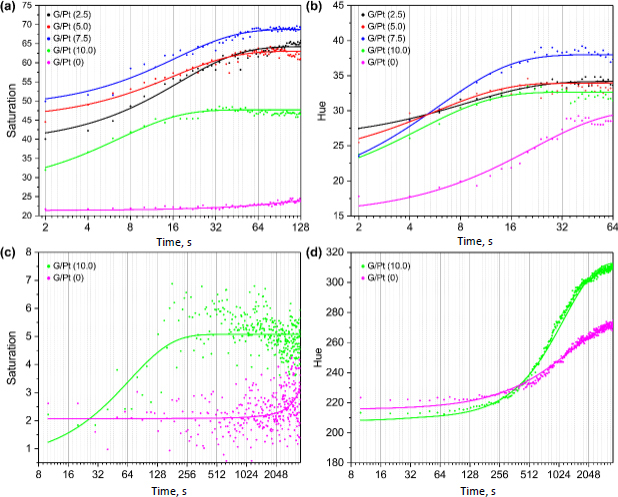
Intensity of colour changes over time of (a) dopamine and (b) NADH using FeCl_3_ + phenanthroline, (c) dopamine using DNP, and (d) NADH using resazurin with the various additions of G/Pt

The colour change produced by reactions occurs exponentially over time for dopamine using FeCl3 and phenanthroline, with a single peak of the highest intensity, as seen in Figure 8(a). Similar behaviour is observed for NADH in Figure 8(b). Compared to colour changes without G/Pt, it is evident that the intensity of colour changes significantly increases with the addition of G/Pt as a catalyst, although the intensities produced varied, and no clear trend of intensity change with increasing concentration. Dopamine detection in the absence of G/Pt takes at least 17 times longer to reach the same colour intensity achieved with G/Pt-modified μPAD, while NADH takes at least 2.4 times. Both biomarkers exhibit smaller time constants (τ) with the addition of G/Pt, indicating that G/Pt accelerates the reactions and functions effectively as a catalyst. The colour changes from the reactions of both dopamine with DNP and NADH with resazurin follows an exponential trend over time, as illustrated in Figures 8(c) and (d). For dopamine using μPAD, the colour change is very subtle, leading to more dispersed results. In contrast, the NADH reaction with resazurin produces a much clearer colour change, which is easily observed. Both sets of results demonstrate a similar pattern when G/Pt is added, where the reaction time accelerates compared to without the addition of G/Pt. The time required to reach the peak intensity is analysed through the time constant, as detailed in Table 1.

**Table 1.** Time constants (*τ*) of dopamine and NADH colourimetric reaction

Reagent	Pt concentration in G/Pt, mM	*τ* / s	
		Dopamine	NADH
FeCl_3_ + Phenanthroline	0.0	306.891	21.193
	2.5	17.761	8.7190
	5.0	16.066	5.5638
	7.5	16.719	5.9603
	10.0	6.689	4.5869
DNP / Resazurin	0.0	971.34	1305.45
	10.0	66.34	979.446

**Figure 9.**
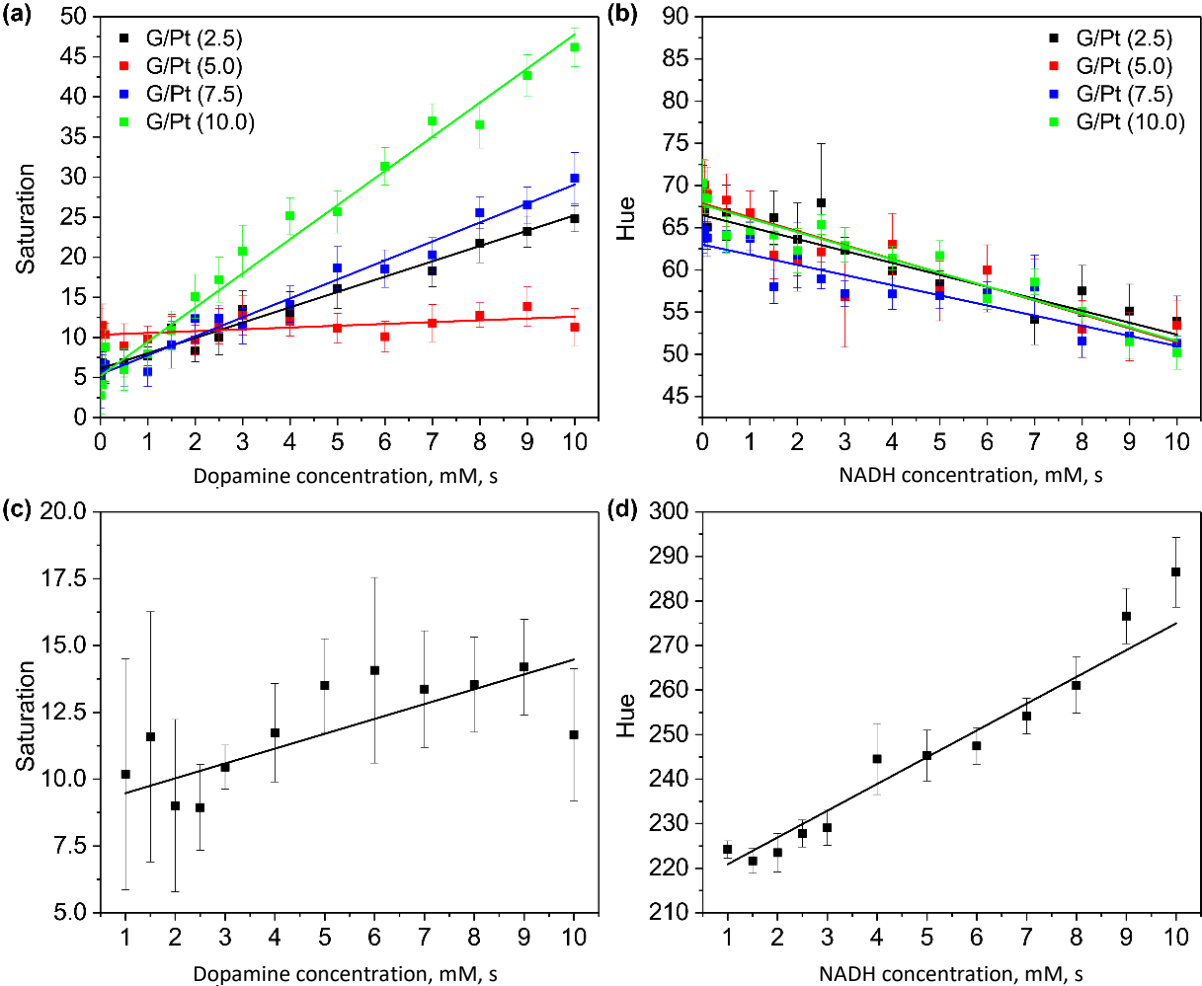
Calibration curve of (a) dopamine and (b) NADH using FeCl_3_ + phenanthroline with the addition of various G/Pt, (c) dopamine using DNP and (d) NADH using resazurin with the addition of G/Pt (10.0)

The calibration curves for dopamine and NADH detection using FeCl_3_ and phenanthroline are obtained as shown in Figure 9(a) and (b).

The dopamine test results show that G/Pt performs relatively well in the saturation channel. G/Pt (10.0) exhibits the best performance in dopamine detection, with a sensitivity value of 4.26 mM^-1^, *R*^2^ of 0.98, LOD of 0.56 mM and LOQ of 1.70 mM. Subsequently, relatively lower linearity values are obtained for the NADH test, with the highest value observed for G/Pt (10.0) on the Hue channel. The LOD and LOQ are relatively large, with the smallest value of 1.20 and 3.63 mM, respectively.

The calibration curve for dopamine using DNP and NADH using resazurin are obtained as shown in Figure 9(c) and (d). Similar to the previous result, dopamine detection shows better results in saturation channels. However, the performance using DNP with the sensitivity of 0.55 mM^-1^, linearity of 0.61, LOD of 4.03 mM and LOQ of 12.20 mM pale in comparison to FeCl_3_ and phenanthroline. This result also shows that DNP is not suitable for quantification as the obtained LOQ value exceeds the linearity range. NADH detection using resazurin, however, shows better results compared to FeCl_3_ and phenanthroline in the hue channel. The detection performance from G/Pt (10.0) exhibits a high sensitivity of 6 mM^-1^ and linearity of 0.94. The LOD and LOQ values also show better results of 0.99 mM and 2.99 mM, respectively.

The multiplex testing to assess reagent selectivity is summarized in Tables 2 and 3. The use of FeCl_3_ and phenanthroline as detection reagents results in similar colour changes for dopamine, NADH, and their mixture, indicating that both compounds can react with the reagent. Quantitatively, dopamine is detected at 9.62 mM, closer to the actual concentration compared to NADH, which is detected at 15.77 mM. However, the similarly high values and indistinguishable colour changes suggest that FeCl_3_ and phenanthroline are not selective in differentiating between dopamine and NADH. In contrast, resazurin produces more distinct colour differences. The reaction with NADH results in a pink colour, consistent with previous findings, while dopamine detection yields a bluish dark gray shade. This gray colour is likely due to the absence of a reaction, leading to a wet paper appearance, combined with the inherent blue colour of resazurin. For the mixture of both compounds, resazurin produces a magenta hue, reflecting the reaction of dissolved NADH, while dopamine remains unreacted. Quantitatively, resazurin detects 8.02 mM of NADH in a 10 mM concentration, providing more accurate results than FeCl_3_ and phenanthroline.

**Table 2.** Detection result of multiplex test

Reagent	10 mM dopamine	10 mM NADH	5 mM dopamine + 5 mM NADH
FeCl_3_ + Phenanthroline	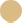	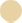	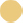
Resazurin	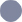	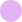	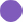
DNP	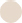	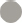	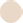

**Table 3.** Quantification result of multiplex test based on HSV colour channel

Reagent	Hue	Saturation	Value	Detected concentration, mM	
				Dopamine	NADH
FeCl_3_ + Phenanthroline	42.27	46.20	92.18	9.62	15.77
Resazurin	263.08	51.38	73.50	-	8.02
DNP	30.94	14.35	91.41	9.77	-

Similar outcomes are observed with DNP, which selectively reacts with dopamine, producing a faint yellow colour, and does not react with NADH, resulting in a gray appearance similar to wet paper. In the presence of both compounds, the colour change is consistent with that of dopamine detection. DNP demonstrates a high level of accuracy, detecting 9.77 mM dopamine from the mixture, comparable to FeCl_3_ and phenanthroline. Overall, FeCl_3_ and phenanthroline demonstrate higher accuracy in detecting dopamine compared to NADH. However, the similar colour produced for both biomarkers suggests limited selectivity, indicating less performance in distinguishing between dopamine and NADH. On the other hand, resazurin and DNP each show good selectivity and reliable detection performance for NADH and dopamine, respectively. Based on these findings, FeCl_3_ and phenanthroline are most suitable for dopamine detection, while resazurin is the preferred reagent for NADH detection. The sensing performance of this G/Pt nanocomposite-based biosensor has been summarized in Table 4 along with those reported in the literature in Table 5.

**Table 4.** Sensing performance of dopamine and NADH detection

Analyte	Reagent	G/Pt content, mM	Sensitivity, mM^-1^	*R* ^2^	LOD, mM	LOQ, mM
Dopamine	FeCl_3_ + Phenanthroline	2.5	1.91	0.97	0.66	1.99
		5.0	0.23	0.32	5.58	16.90
		7.5	2.37	0.97	0.66	2.00
		10.0	4.26	0.98	0.56	1.70
	DNP	10.0	0.55	0.61	4.03	12.20
NADH	FeCl_3_ + Phenanthroline	2.5	1.42	0.90	1.40	4.23
		5.0	1.64	0.87	1.62	4.92
		7.5	1.20	0.91	1.69	5.12
		10	1.61	0.92	1.20	3.63
	Resazurin	10	6.00	0.94	0.99	2.99

**Table 5.** Comparison with other paper-based biosensors with various reagents

Analyte	Reagent	Linear range, mM	LOD, μM	Reference
Dopamine	FeCl_3_ + Phenanthroline	0.52×10^-3^ to 4.75×10^-3^	0.37	[30]
	hydrogen peroxide, starch, and sulfuric acid	0.01 to 1.0	5	[33]
	Graphene quantum dots	0.025 to 0.075	25	[34]
	ZIF-67 MOF + 4-AAP	0.01 to 1.0	2.75	[35]
	Iodine + amylose	0.01 to 10	10	[36]
	FeCl_3_ + Phenanthroline	0.01 to 10	560	This work
	2,4-DNP	1 to 10	4,030	
NADH	G-Pd + H_2_O_2_	0.1 to 0.6	-	[18]
	Ammonium iron (III) sulfate	0.045 to 7.5	45	[37]
	FeCl_3_ + Phenanthroline	0.01 to 10	1,200	This work
	Resazurin	1 to 10	990	

### Smartphone application test

An Android application has been successfully developed for easier automatic detection of biomarkers, using the level 21 API and the OpenCV Android module (details in Supplementary material). The application has been tested on Samsung Galaxy M23 and Redmi Note 11 smartphones and runs without major issues. Small issues regarding layout are present that fail to adapt to various device screen sizes. Nevertheless, the application can capture μPAD images, process them, and display the concentration readings. The Mean Absolute Percentage Error (MAPE) testing for the smartphone application is conducted using 6 variations outside the calibration curve to assess the sensor's accuracy. Based on the MAPE test results as shown in Table 6, it is found that the values obtained are not significantly different (less than 1 percent) from the manual measurements made with ImageJ. This indicates that the algorithms, particularly in the thresholding and masking stages, yield good results in determining the detection zones and obtaining colour values. The best values are obtained for dopamine with G/Pt (2.5) in the saturation channel and NADH with G/Pt (7.5) in the hue channel, both of which are consistent with the results from manual testing.

**Table 6.** MAPE summary of multiplex test

Analyte	G/Pt (mM)	MAPE, %	
		Manual	Automatic
Dopamine	2.5	0.12	0.26
	5.0	0.28	0.37
	7.5	0.16	0.44
	10.0	0.52	1.05
NADH	2.5	0.06	0.04
	5.0	0.05	0.05
	7.5	0.03	0.05
	10.0	0.07	0.04

## Conclusions

A biomarker detection kit has been successfully developed with a 3D origami μPAD. The catalyst, G/Pt nanocomposites, is successfully synthesized using a photocatalytic reduction method. The addition of G/Pt as a catalyst accelerates the reactions and increases the colour intensity in colourimetric detection. The best overall performance for biomarker detection is obtained with G/Pt (10.0), i.e., the use of 10 mM H2PtCl6 as the Pt precursor. Nonetheless, the aggregation of Pt nanoparticles on the graphene matrix could not be hindered, and a future synthetic strategy by photochemical reduction to avoid Pt nanoparticle aggregation can be developed. The selectivity of reagent combinations is confirmed by multiplex testing. An Android application has also been successfully developed, enabling the detection of biomarkers through captured μPAD images with the assistance of a fabricated detection chamber. The biosensor exhibits good linearity and acceptable performance in detecting dopamine and NADH, comparable to other non-origami paper-based biosensors. Therefore, the results at hand could pave the way for the development of an affordable, portable, and easy-to-use diagnostic kit. Further, the utilization of the 3D origami microfluidic structure alongside smartphone-assisted analysis provides a flexible and scalable framework for point-of-care diagnostics and personalized health monitoring.


