# Supplementary material for: Monolayer graphene/platinum-modified 3D origami microfluidic paper-based biosensor for smartphone-assisted biomarkers detection
Source: ADMET DMPK. 2025 Jul 20;13(4):2833. doi: 10.5599/admet.2833 (PMC12335305; doi:10.5599/admet.2833)
Supplement: Supplementary file 1 [file ADMET-13-2833-S1.pdf]

ADMET & DMPK 13(4) (2025) S2833

Open Access : ISSN : 1848-7718

http://www.pub.iapchem.org/ojs/index.php/admet/index

Supplementary material to

Monolayer graphene/platinum-modified 3D origami microfluidic paper-based biosensor for smartphone-assisted biomarkers detection

Arda Fridua Putra[1], Annisa Septyana Ningrum[1], Suyanto[1], Vania Mitha Pratiwi[2], Muhammad Yusuf Hakim Widianto[3], Irkham[4], Wulan Tri Wahyuni[5], Isnaini Rahmawati[6], Fu-Ming Wang[7,8], Chi-Hsien Huang[1,9] and Ruri Agung Wahyuono[1]

Department of Engineering Physics, Institut Teknologi Sepuluh Nopember, Surabaya 60111, Indonesia
[2]Department of Materials Engineering, Institut Teknologi Sepuluh Nopember, Surabaya 60111, Indonesia
[3]Department of Mathematics, Institut Teknologi Sepuluh Nopember, Surabaya 60111, Indonesia
[4]Department of Chemistry, University of Padjadjaran, Sumedang 45363, Indonesia
[5]Department of Chemistry, Institut Pertanian Bogor (IPB) University, Bogor 16680, Indonesia
[6]Department of Chemistry, University of Indonesia, Depok 16424, Indonesia
[7]Graduate Institute of Applied Science and Technology, National Taiwan University of Science and Technology, Taipei 10607, Taiwan
[8]Graduate Institute of Energy and Sustainability Technology, National Taiwan University of Science and Technology, Taipei 10607, Taiwan
[9]Department of Materials Engineering, Ming Chi University of Technology, New Taipei City 24031, Taiwan

ADMET & DMPK **13(4)** (2025) 2833; https://doi.org/10.5599/admet.2833

Camera optimization

Camera optimization was conducted to ensure consistency between captured and original images with configurations of ISO values (200, 400, and 640) and shutter speeds (1/60 and 1/45). The sample image captured using the optimized configuration is shown in Figure S1 and all condition during optimization is summarized in Table S1.

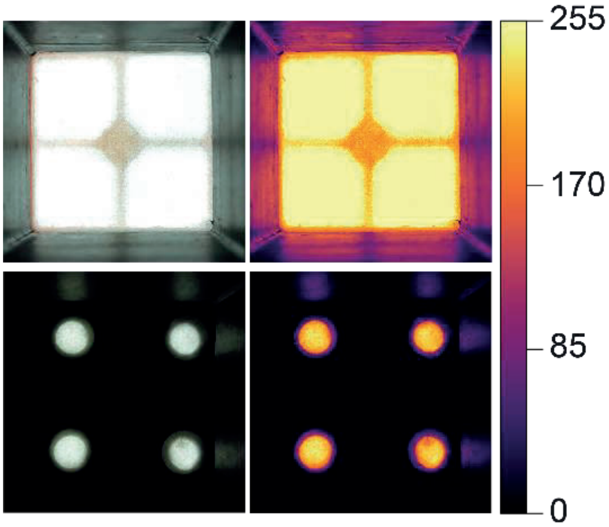

Figure S1. Sample image captured using focus 0.1, ISO 200, shutter speed 1/60 and white balance (WB) 4400K, and the heatmap analysis

Table S1. Grayscale value of sample images taken using various camera setting

Shutter speed	Intensity					Shutter speed	Intensity				
	ISO	Average	Min	Max	Std Dev		ISO	Average	Min	Max	Std Dev
1/60	200	176.35	67	223	27.18	1/20	200	216.64	77	255	40.01
	400	216.97	107	251	24.81		400	241.04	110	255	26.34
	640	224.18	77	255	38.94		640	248.29	162	255	15.63
	800	231.84	79	255	35.84		800	249.68	167	255	13.48
	1600	247.66	160	255	16.51		1600	253.17	191	255	6.30
	3200	250.01	82	255	15.52		3200	254.09	154	255	4.34
1/45	200	186.65	73	234	27.56	1/10	200	240.25	123	255	26.78
	400	218.70	85	253	32.68		400	250.21	151	255	13.21
	640	230.91	89	255	36.28		640	253.17	202	255	6.38
	800	239.69	112	255	26.92		800	253.08	193	255	6.65
	1600	249.02	153	255	14.65		1600	254.76	232	255	1.17
	3200	251.59	140	255	10.14		3200	254.71	184	255	2.76

RGB analysis

After capturing images through smartphone, RGB analysis was performed using in-built RGB Measure plug-in within ImageJ. ROIs were selected using oval tool manually for each circle.

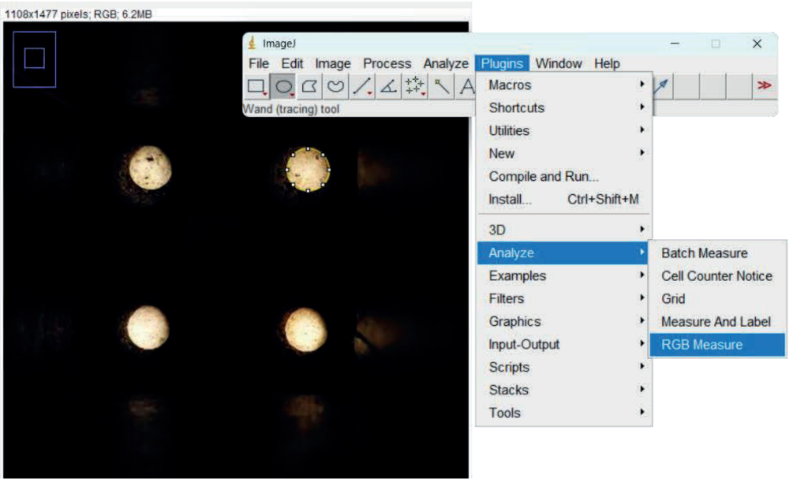

Figure S2. Measurement of RGB using open source software of ImageJ

The obtained RGB values were then converted to HSV using Equations (S1) to (S3):

$$H=\begin{cases}\dfrac{\left(\dfrac{G-B}{max_R-min_R}+0\right)}{6}, & \text{if } max=R^{*}\\ \dfrac{\left(\dfrac{B-R}{max_G-min_G}+2\right)}{6}, & \text{if } max=G\\ \dfrac{\left(\dfrac{R-G}{max_B-min_B}+4\right)}{6}, & \text{if } max=B\end{cases} \qquad (S1)$$

*if H < 0, H = 1

$$S=\begin{cases}\dfrac{max_R-min_R}{max_R}, & \text{if } max=R\\ \dfrac{max_G-min_G}{max_G}, & \text{if } max=G\\ \dfrac{max_B-min_B}{max_B}, & \text{if } max=B\end{cases} \qquad (S2)$$

$$V = max_{RGB} \qquad (S3)$$

CC BY

RGB and HSV values were then plotted using Origin, fitted using linear equations to obtain linearity, slope/gradient, and standard deviation of y-intercepts. LOD and LOQ were then calculated using Equations (S4) and (S5):

$$LOD=3.3\frac{StdDev}{Slope} \tag{S4}$$

$$LOQ=10\frac{StdDev}{Slope} \tag{S5}$$

Image processing line code

The image processing algorithm was constructed using Phyton 3 OpenCV module with the following base code:

```
#-----Import modules-----#
import numpy as np
import cv2 as cv
import math
import os
import pandas as pd
from google.colab.patches import cv2_imshow

#-----Import image-----#
img = cv.imread('Selected.jpg')
x,y=img.shape[1],img.shape[0]

#-----Duplicate image and apply filter-----#
imgcrop = img.copy()
gray = cv.cvtColor(imgcrop, cv.COLOR_BGR2GRAY)
blurred = cv.GaussianBlur(gray, (5, 5), 0)

#-----Detect circles using HoughCircles-----#
circles = cv.HoughCircles(blurred, cv.HOUGH_GRADIENT, 1, 20, param1=50, param2=30, minRadius=10, maxRadius=30)
circles = np.uint16(np.around(circles))
x_coords = []
y_coords = []
for i in circles[0, :]:
  # Draw the outer circle
  cv.circle(imgcrop, (i[0], i[1]), i[2], (0, 255, 0), 2)
  # Draw the center of the circle
  cv.circle(imgcrop, (i[0], i[1]), 2, (0, 0, 255), 3)
  x_coords.append(i[0])
  y_coords.append(i[1])

min_x = min(x_coords)
max_x = max(x_coords)
min_y = min(y_coords)
max_y = max(y_coords)

#-----Calculate croppng region-----#
side_length = max(max_x - min_x, max_y - min_y)
center_x = (min_x + max_x) // 2
center_y = (min_y + max_y) // 2
start_x = int(center_x - side_length // 2)
start_y = int(center_y - side_length // 2)

start_x = max(0, start_x - side_length//4)
start_y = max(0, start_y - side_length//4)
end_x = min(x, start_x + side_length + side_length//2 )
end_y = min(y, start_y + side_length + side_length//2 )

#-----Crop the image-----#
cropped_img = img[start_y:end_y, start_x:end_x]
```

```
#-----Redetect circles from the cropped image-----#
imgload = cropped_img.copy()
gray = cv.cvtColor(imgload, cv.COLOR_BGR2GRAY)
blurred = cv.GaussianBlur(gray, (5, 5), 0)
circles = cv.HoughCircles(blurred, cv.HOUGH_GRADIENT, 1, 20, param1=50, param2=30, minRadius=10, maxRadius=30)
circles = np.uint16(np.around(circles))

#-----Sort circles based on their coordinate-----#
currentdata = []
sorted_circles = sorted(circles[0, :], key=lambda circle: (circle[1], circle[0]))
for i, circle in enumerate(sorted_circles):
  center_x = int(circle[0])
  center_y = int(circle[1])
  radius = int(circle[2])

#-----Extract RGB and HSV color from each circle-----#
  bgr_color = imgload[center_y, center_x]
  b, g, r = bgr_color

  hsv_color = cv.cvtColor(np.uint8([[bgr_color]]), cv.COLOR_BGR2HSV)[0][0]
  h, s, v = hsv_color

  currentdata.append([i, r, g, b, h, s, v])

#-----Display result and save as CSV-----#
print(currentdata)
df=pd.DataFrame(currentdata, columns=['Circle', 'R', 'G', 'B', 'H', 'S', 'V'])
df.to_csv('colorimetryreading.csv', index=False)
```

Android spplication

A simple application depicted in Figure S3 was developed to facilitate easier image processing through capturing or importing images and showing the results with additional operating instructions. Following GitHub link can be accessed to clone the application: https://github.com/SilverMane44/MiSense.

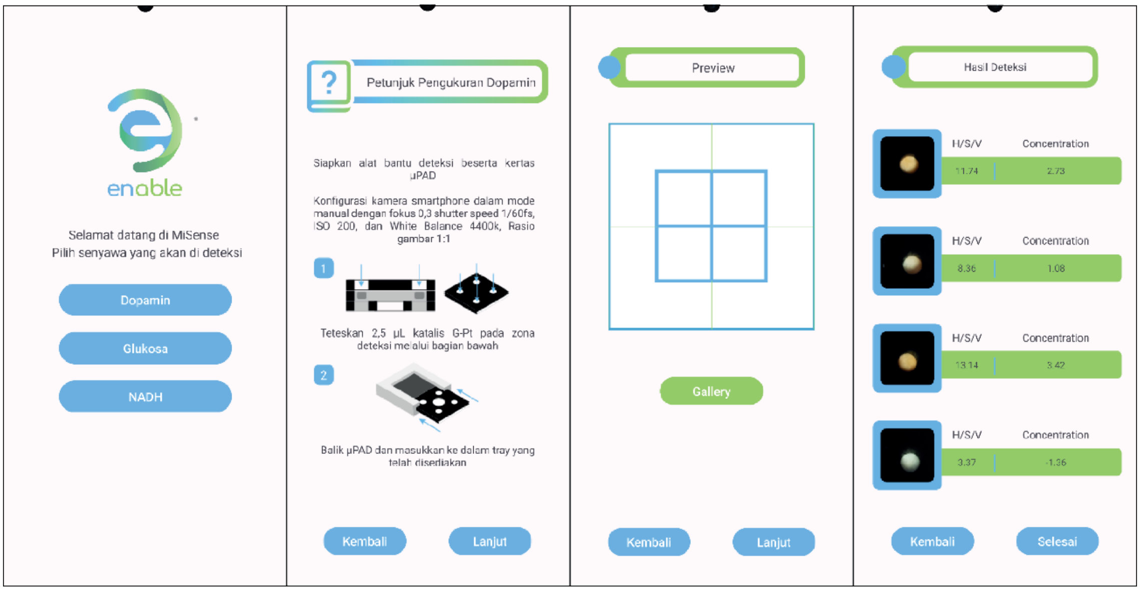

Figure S3. Preview of the developed android application for smartphone measurement

Computational model

Three different interactions of Pt on graphene matrix for simulation are shown in Figure S4. The absorption and extinction coefficient spectrum of each graphene/Pt interaction are shown in Figure S5. These absorption spectra are used to be validated with experimental spectra.

(cc) BY

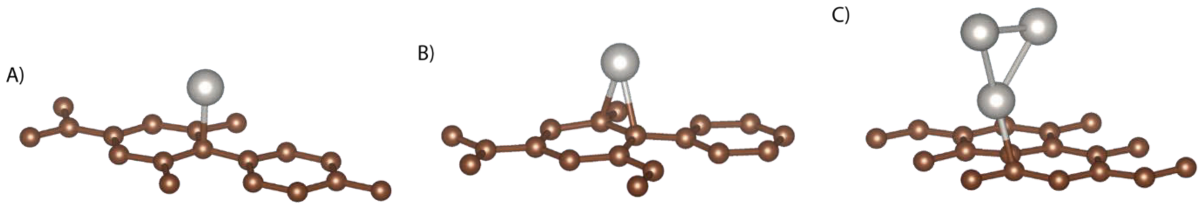

Figure S4. a) a single Pt atom at the top of single C atom of graphene layer. b) a single Pt atom at the bridge C-C of graphene layer. c) Three Pt atoms at the graphene layer. Silver and brown colors indicate the Pt and C atoms, respectively

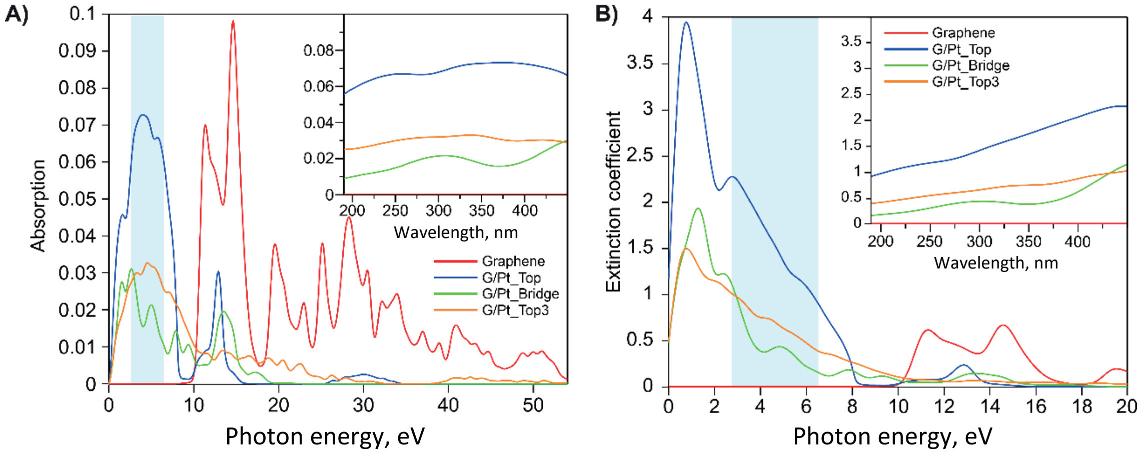

Figure S5. A) Absorption and B) extinction spectra of various G/Pt from computational models
